# An aerial spot-spraying technique: a pilot study to test a method for pest eradication in urban environments

**DOI:** 10.1186/2193-1801-3-750

**Published:** 2014-12-18

**Authors:** Tara M Strand, Carol A Rolando, Brian Richardson, Stefan Gous, Martin KF Bader, Don Hammond

**Affiliations:** Scion, Crown Research Institute, Te Papa Tipu Innovation Park, 49 Sala Street, Rotorua, 3010 New Zealand; Hammond Resource Management Ltd, PO Box 1035, Rotorua, 3040 New Zealand

**Keywords:** Aerial application, Efficacy, Eradication, Deposition, Spot-spray

## Abstract

**Background:**

Pest eradication strategies that use pesticides require application methods that have the lowest environmental and human health impact while maintaining the highest probability of success. This is highly important when eradication takes place in sensitive areas, such as urban or riparian zones. A new aerial application method, the spot-gun, was developed to provide good pesticide coverage on host species while minimising off-target exposure. This type of targeted aerial approach is useful in areas where conventional broadcast aerial spraying was historically used but was not ideal due to the quantity of pesticide hitting non-host species and going off-target. An even distribution of the active component is essential for eradication.

**Findings:**

The spot-gun method was tested and found to provide an even distribution of dye on the adaxial and abaxial leaf surfaces as well as in the top and middle regions in both the inner and outer portions of the canopy. The form of the deposits on the leaf surface was very different from that obtained using a broadcast aerial application with a conventional spray boom.

**Conclusions:**

The distribution results imply that the spot-gun method treatment will provide good efficacy. The implications of the different deposit pattern on efficacy are not known at this stage. This aerial spot-spray method has considerable potential as a tool for targeted aerial application of pesticides to small areas of difficult to reach canopies.

**Electronic supplementary material:**

The online version of this article (doi:10.1186/2193-1801-3-750) contains supplementary material, which is available to authorized users.

## Background

Cost-effective eradication of potentially damaging pests often requires the use of aerially applied pesticides and other formulations (Hosking et al. 2003). In situations where insect pests are targeted by applying pesticide to foliage that is subsequently ingested, treatment efficacy relies upon application methods that provide consistent leaf surface coverage and ensures that target organisms have a high probability of receiving a lethal dose within the treated area (Richardson et al. 2005). In eradication programmes where the target organism is distributed over large areas or located in tall trees, the conventional ‘broadcast’ aerial application provides the most cost-effective means of pesticide delivery (Richardson 2002).

Conventional broadcast aerial application becomes complex and often undesirable when the eradication programme is near urban centres or environmentally sensitive regions (Richardson and Thistle 2002). While risks can be minimised by applying the lowest dose of pesticide necessary for eradication (Brockerhoff et al. 2010) treatment of large areas cannot be avoided with the standard broadcast application even when the pest targeted for eradication is limited to a small area. Under these circumstances, novel methods and technologies are needed to support ongoing use of aerial application methods for eradication and/or management of pests, particularly when the pests are located in or near sensitive environments and urban centres (Suckling et al. 2013). The concept of a targeted aerial application for situations where pest distributions are limited to small areas has been previously trailed. Examples include the spray ball and pyramid investigated for herbicide use in south-western United States by Throop et al. (2013) and a targeted aerial spraying method evaluated by Richardson (2002) for release of the biocontrol agent *Bacillus thuringiensis* var. *kurstaki* (Btk). The spray ball and pyramid methods were found to work well for targeting specific plant species while minimising off-target effects, however due to the economic costs associated with these methods they were recommended for use in difficult terrain or in highly sensitive environments. The targeted method evaluated by Richardson (2002) was demonstrated to be ineffective due to the operational constraints of a high release height combined with use of small droplets (for efficacy). The conclusion from that study supported a conventional broadcast boom application and subsequent large buffer zones to ensure a high probability that the targeted area received a lethal dose. In other words, the goal of only applying pesticide to the area with the pest infestation was not achieved. The aim of this paper is to present a new aerial pesticide application method that achieves low spray drift but good foliar coverage designed for situations where pests are restricted to small areas or a few individual trees.

The new methodology builds on the aerial ‘spot-gun’ technology that was previously used in New Zealand to aerially apply systemic herbicides to isolated, wild non-native pines. To date, the spot-gun method has only been used to deliver systemic herbicides, which do not require the degree of canopy coverage essential for typical insecticides used in eradication operations. For the aerial spot-gun method to be acceptable in a pest eradication programme, it must deliver, at the minimum, the same level of insecticide coverage and distribution throughout the canopy as that provided by a conventional broadcast application.

A prototype spot-gun for targeted aerial application of insecticides was developed and tested through a pilot trial. This paper presents the spot-gun prototype and method, as well as the pilot trial results. The aim of the study was to examine the relative differences between the spot-gun application and the conventional broadcast application in terms of the distribution of deposits along vertical and horizontal canopy profiles and on the adaxial and abaxial surfaces of leaves.

## Materials and methods

### Trial site and description

A trial was conducted in a mature canopy of *Eucalyptus nitens* (Deane and Maiden) Maiden on a relatively flat hilltop near Rotorua, New Zealand. The trees were approximately 12 years old and on average 23 m tall with an average diameter at 1.4 m above the ground of 30 cm. The live canopy began approximately 13.7 m above ground level and had an average depth of 9.4 m. A meteorological tower was deployed near the trial site and recorded winds, temperature and relative humidity (Monitor Sensors, Caboolture, Queensland Australia) at 1.8 m above ground level (AGL). The tower was located in an open field approximately 400 m from the trial location. The data represent the conditions found above the canopy. The average temperature, relative humidity, and wind speed for the duration of the trial were 9.5°C, 64.2%, and 2.21 m s^−1^, respectively.

### Treatments

Two aerial spray application treatments were used in the trial: broadcast (using a conventional boom) and spot-gun. The treatment areas were 100 m apart and spaced so that wind did not drift spray from one treatment to the other. To further ensure against cross-treatment contamination, the conventional broadcast treatment was only applied after the sample trees in the spot-gun treatment had been felled. For each treatment the sample trees were randomly selected from within the treatment area.

The applied spray solution was a mixture of the Yellow Fluorescent Pigment SC (Topline Paint Pty Ltd, Lonsdale, South Australia) at 1% v/v. and 0.1% Pulse (Nufarm, Otahuhu, Auckland, New Zealand) in water. All treatments were applied using a Bell JetRanger helicopter with a 10.16 m main rotor diameter fitted with a GPS (Flagman, Del Norte Technology Limited, Doncaster UK). The helicopter flew at a nominal height of approximately 5 m above the canopy.

#### Spot-gun application

A custom built spot-gun comprising of two D14-46 nozzles at the end of a 2 m long lance with a pressure gauge was designed to deliver 8.25 ℓ/min at 200 kPa pressure when triggered by hand. The spot-gun produced a droplet size spectrum classified as ‘very coarse’ (>500 μm volume median diameter, VMD). Spray was applied to ten randomly selected individual trees by hovering the helicopter above the tree canopy while the spray solution was applied for fifteen seconds, delivering two litres of spray solution per tree. This rate was selected to maximise foliar coverage and to test the spot-gun’s capabilities to evenly distribute dye throughout the canopy. After the application of this treatment, the spray deposits were allowed to dry before five trees were selected from ten sprayed trees and felled to enable sampling of leaves throughout the canopy.

#### Conventional broadcast application

All nozzles were positioned straight back and evenly distributed within 40% of the rotor diameter; specifics of the boom setup are shown in Table 1. The broadcast application was accomplished by flying ten swaths with 4 m lane separation (distance between flight lines). Each swath was approximately 150 m long, and the average helicopter velocity was 26 knots (13.4 m s^−1^). The aircraft was calibrated to deliver 200 ℓ/ha of spray solution over the treated area (0.6 ha). After the application of the treatment the spray deposits were allowed to dry and then five trees were randomly selected from the treated area and felled to enable sampling of leaves throughout the canopy.

**Table 1:** **Description of conventional broadcast aerial application set up on a Bell JetRanger helicopter with nominal droplet size and the nozzle type, number and configuration used to achieve the droplet size range, and the resulting application rates**

Standard application parameter	Parameter value
Nominal droplet size (μm VMD^a^)	255
Nozzle Type^b^	TeeJet 8005 (flat fan)
Number of nozzles	31
Configuration of nozzles on boom^c^	10, 11, 10
Flow rate per nozzle (ℓ/min)	2
Pressure (bar)	2.5
Flow rate (ℓ/min)	62
Application rate (ℓ/ha)	200

^a^VMD is volume mean diameter.

^b^All nozzles were manufactured by Spray Systems Co, Wheaton IL, USA.

^c^left, centre, and right; Nozzles spaced together without blanks at centre of aircraft.

### Description of crown sampling

Once the sample trees were on the ground the crown samples were collected and basic measurements of tree dimensions were made. The tree dimensions measured include: i) total tree length/height (m); ii) canopy length (m), defined as the top of tree to bottom of green foliage; iii) diameter of stem at breast height (cm) and; iv) distance from the top of each tree to each branch sampled (m). A stratified sampling protocol ensured leaves were collected throughout the canopy, although leaves touching the ground were not sampled. Leaves were collected along the length (vertical profile) of the live canopy from both the outer and inner (horizontal profile) portions providing two deposition profiles. Ten leaves made up a sample and were collected at various points moving from the top of the crown to the bottom. The top of the tree was defined as the first 1 m of fully expanded foliage. Three samples of 10 leaves were collected from the top region of the tree. Moving down from the top 1 m, for every branch that was not on the ground, a sample of ten leaves was taken from near the tree trunk, or inner portion of the canopy, and from the end of the branch, or outer portion of the canopy.

### Assessment of spray coverage

An assessment of fluorescent dye coverage on the sampled leaves was made by visual inspection of the leaf while holding under an ultraviolet light. Both the adaxial and abaxial surfaces of the leaves were examined for spray coverage. Deposition as spray coverage of the leaf was ranked (binned) in 10% increments from 0%, for no visible coverage, to 100%, for full coverage of the leaf. The spray coverage was also scored for the shape of the droplet deposited on the leaf surface as either ovoid or a smear (no definable shape).

### Statistical analysis

The semi-quantitative binned coverage data were used in a statistical analysis to assess foliage coverage throughout the crown, both vertically and horizontally, for both treatments. A cumulative link mixed model fitted with Laplace approximation and flexible thresholds (R-package ‘ordinal’, Christensen 2013) was applied to test the dependence of the ordinal response variable leaf coverage (on an interval scale from 0 to 100%) on spraying methodology. Leaf coverage was assessed along the vertical profile (top, middle, and bottom) and the horizontal profile (inner and outer). Furthermore, leaf coverage was assessed on both the adaxial and abaxial leaf surface. The crown subsampling routine was accounted for by incorporating a random term with the following nesting structure: ‘leaf surface’ in ‘horizontal crown region’ in ‘vertical crown region’ in ‘tree individual’ (five trees per treatment). A variance components analysis was applied to estimate the contribution of each random effect to the variance of the response variable. All statistical analyses were conducted using R version 3.0.1 (R Core Team 2013)

## Results

Deposition results from the two aerially applied treatments, conventional broadcast and spot-gun, were compared to evaluate the spot-gun’s capability to uniformly distribute active ingredient (dye) horizontally and vertically through the canopy and to cover the adaxial and abaxial leaf surfaces. The application method significantly influenced the dye coverage found on the leaves (Table 2). Statistically, nearly 100% of the total variance was explained by leaf position in the vertical and horizontal crown zones (Table 2).

**Table 2:** **Results from a cumulative link mixed model testing the effect of the conventional broadcast and spot-gun applications deposition of dye on the adaxial and abaxial leaf surfaces**

Parameter	***Estimate***	***SE***	Z	***P***
Treatment	4.79	1.31	3.65	<0.001***
Random effect		Variance (%)		
Tree		<0.1		
Vertical crown region		48.36		
Horizontal crown region		51.63		
Adaxial/abaxial surface		<0.1		

Signif. codes: 0 ‘***’0.001 ‘**’0.01 ‘*’0.05.

Variance components derived from the cumulative link mixed model are also shown.

### Distribution of deposit on the adaxial leaf surfaces

**Figure 1:**
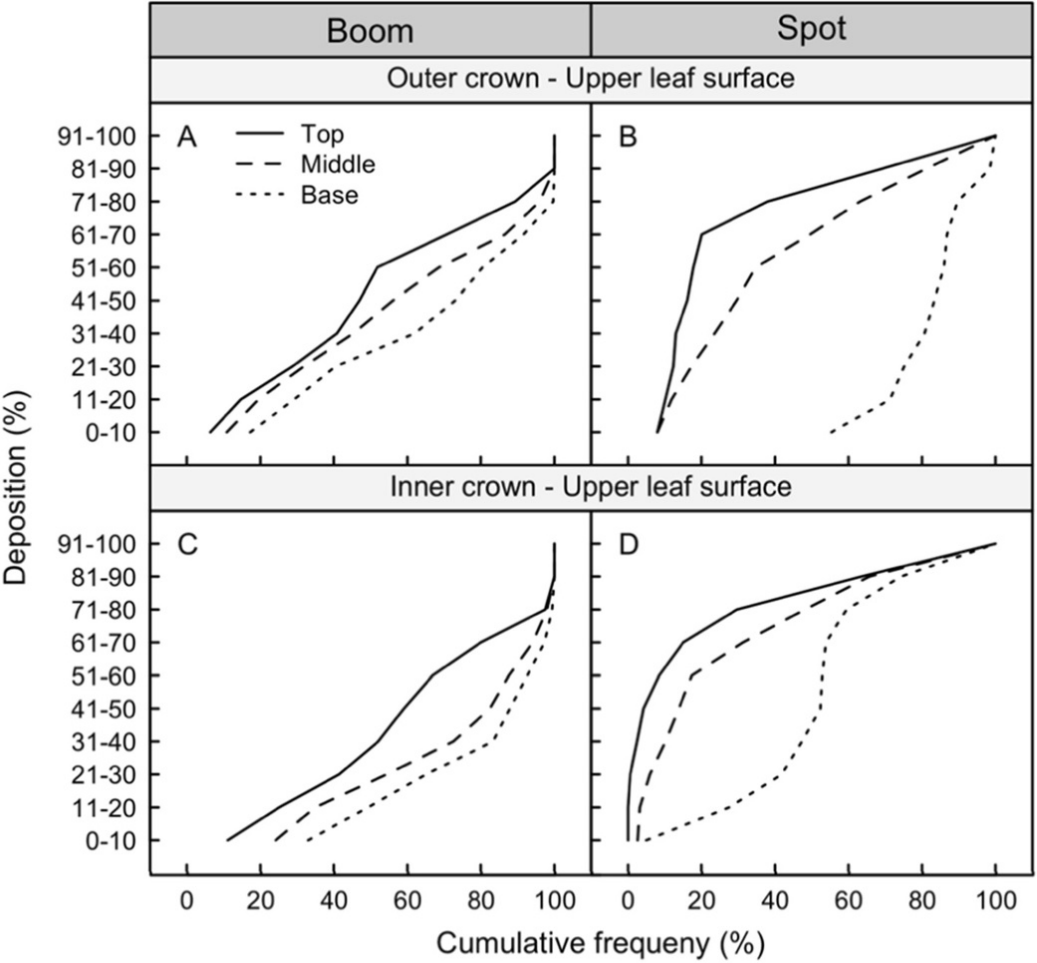
**Cumulative frequency curves of deposition found on the adaxial (upper) leaf surfaces for the conventional broadcast (panels A and C) and spot-gun (panels B and D) treatments.** If the cumulative frequency curves are similar in magnitude and shape then the distribution of dye was similar.

The spot-gun application distributed the dye evenly in the top and middle regions of the canopy in both the inner and outer regions and this is expressed as similar shaped and somewhat overlapping lines (Figure 1B and D). The base of the canopy received lower dye coverage compared to the middle and the top and the inner-base region received more dye than the outer-base region of the canopy.

The conventional broadcast application provided an even distribution of dye throughout the vertical profile of the outer canopy (Figure 1A). In the inner portion of the canopy, the middle and base regions show similar deposited dye coverage to each other, but are lower than the top of the canopy and the outer portion of the canopy (Figure 1C).

### Distribution of deposit on abaxial leaf surfaces

**Figure 2:**
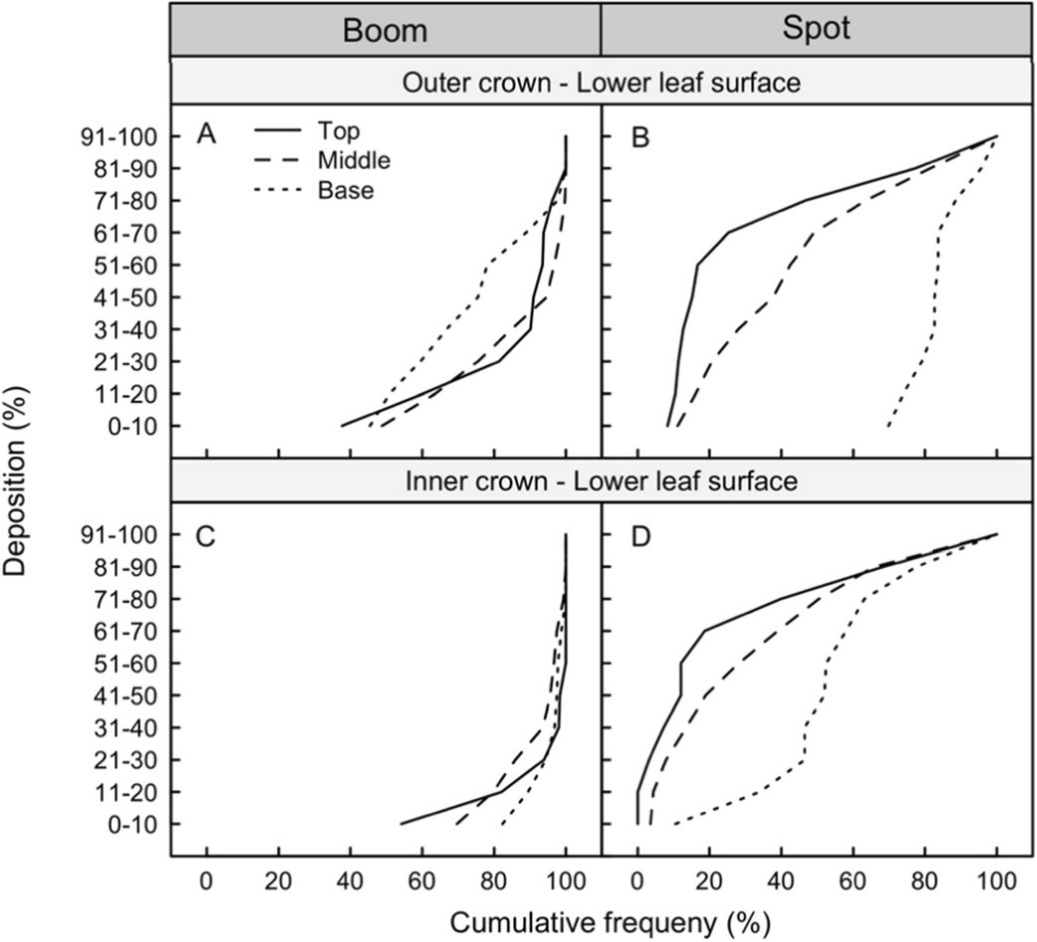
**Cumulative frequency curves of deposition found on the abaxial (lower) leaf surfaces for the conventional broadcast (panels A and C) and spot-gun (panels B and D) treatments.** If the cumulative frequency curves are similar in magnitude and shape then the distribution of dye was similar.

**Figure 3:**
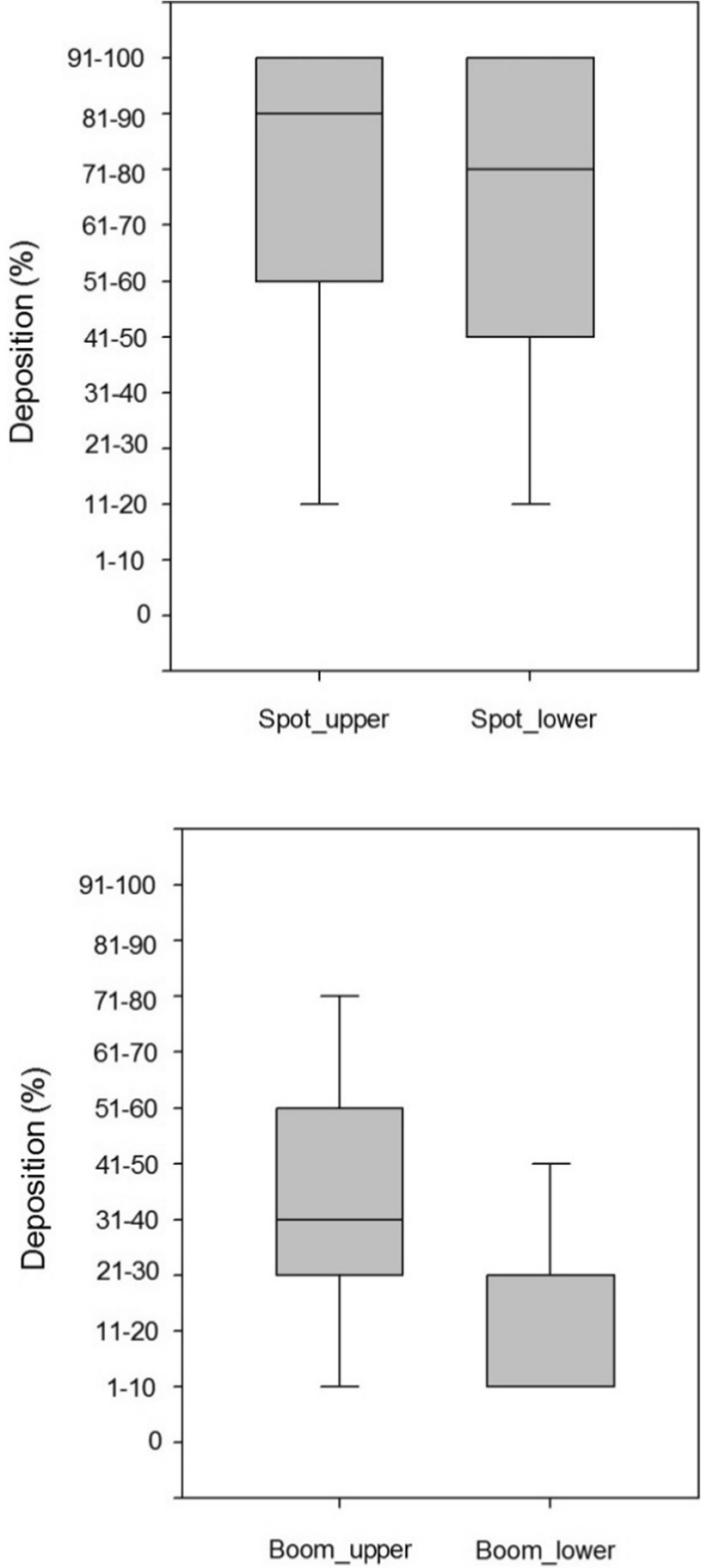
**Box plots of the deposited dye, visually assessed into 10% bins, found on upper (adaxial) and lower (abaxial) leaf surfaces demonstrate that the spot-gun application (top) provided similar coverage on both leaf surfaces whereas the broadcast application (bottom) did not.** Each treatment had different application rates, therefore a one-to-one comparison across treatments cannot be made. The line in the centre of the boxes shows the median value, while the first and third quartiles are the bottom and top boundaries of the box. The whiskers extend to the minimum and maximum values. The data were bound by the upper limit of the 91-100% bin and the lower limit of the 1-10% bin.

The spot gun application provided a similar distribution of deposited dye on both the adaxial and abaxial leaf surfaces (Figures 2B and D versus 1B and D). The spot-gun application provided similar deposition of dye on the bottom of the leaf surfaces compared to the top of the leaf surfaces (Figure 3). Median values of deposition were in the 81-90% leaf coverage range for the adaxial leaf surfaces and the 71-80% range for the abaxial leaf surfaces. For the conventional broadcast treatment, the distribution of dye on the abaxial leaf surfaces varied from the adaxial leaf surfaces, with the abaxial leaf surfaces receiving less dye than the adaxial leaf surfaces (Figures 2A and C versus to 1A and C). The conventional broadcast method poorly covered the abaxial leaf surfaces with a median value in the 1-10% range for the abaxial leaf surfaces, compared to a median in the 1-40% range for the adaxial leaf surfaces (Figure 3).

## Discussion

The relative difference between deposited dye on the adaxial and abaxial leaf surfaces is the key result, not the difference in magnitude between application methods (Figure 3). The spot-gun method produced very little difference in deposited dye found on the abaxial leaf surface compared to the adaxial leaf surface. Conversely, the difference in deposited dye between the adaxial and abaxial leaf surfaces sampled from the conventional broadcast application was large. This demonstrates the difficulty that the conventional broadcast method has of covering the undersides of leaves.

The overall deposition of dye was higher on the leaves sampled from the spot-gun method and this was due to the difference in application rates of the spray solution. The spot-gun application used a higher rate of solution per tree compared to the conventional broadcast application. The application rate for the conventional broadcast application was a standard rate while the spot-gun application rate was selected based on providing maximum coverage possible in order to test the spot-gun’s capabilities. Future work will include optimisation of the spot-gun with lower application rates.

**Figure 4:**
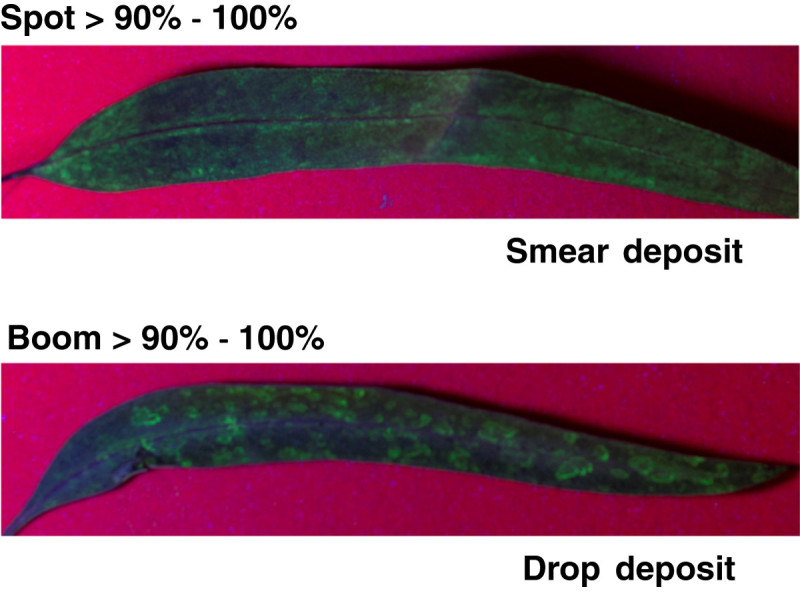
**Example of the ‘smear’ deposit produced by the spot-gun application (top) and, for comparison, the ovoid deposit produced by the standard broadcast method (bottom).** Both leaves were placed in the 90%-100% coverage bin.

An interesting difference between the two aerially applied methods was the form of the deposit on the leaf surface. The majority of the deposits from the spot-gun method were in the form of a ‘smear’ that spread across the leaf surface with no characteristic droplet ovoid shape (Figure 4). In contrast, deposit forms from the conventional broadcast application were found to be round and ovoid in shape. The smear provided a higher percentage of leaf coverage and high coverage leads to good treatment efficacy. The difference in deposit form could play a significant role in the quantity of active ingredient within each deposit. This may affect the efficacy of the treatment and further investigation is required to quantify the typical dose of active ingredient within a smear deposit.

For eradication it is important to evenly distribute a lethal dose of active ingredient throughout the host canopy, thus minimising pest migration (and survival) to areas of lower doses. This is why the conventional broadcast application, ideally with fine droplets (volume median diameter (VMD) of 150 μm or less), is an effective method for eradication (Richardson 2002; Richardson and Kimberley 2010). Unfortunately, minimising off-target drift usually requires replacing the known effective fine droplet size (150 μm VMD) with a larger droplet size (250+ μm VMD), which compromises the efficacy of the treatment with less coverage, although evenly distributed. This is demonstrated when comparing the distribution of dye between the inner and outer portions of the canopy (Figure 1A and C) and between the adaxial and abaxial leaf surfaces (Figures 1 and 2).

The spot-gun method uses a very coarse droplet spectrum (500+ VMD) and the helicopter’s downwash to distribute the spray droplets down through the canopy. The large droplets minimise drift and other off-target impacts while the downwash provides the coverage and even distribution necessary for treatment efficacy. As expected, with a coarse droplet spectrum, there is less material at the base of the canopy compared to the middle and the top (Figures 1 and 2). Unexpectedly, the distributions of dye on the adaxial and abaxial leaf surfaces were similar throughout the canopy profile, resulting in better coverage on the abaxial leaf surfaces at the base of the inner canopy. The high coverage of dye on the abaxial leaf surfaces was possible due to a helicopter downwash effect, which moved the leaves and exposed both sides of the leaf surfaces to the spray.

We hypothesise that the difference in the distribution of deposited dye at the base of the canopy (compared to the top and middle regions) was due to the angle of the spot-gun relative to the helicopter downwash. Further study could verify this hypothesis and also optimise the method, with respect to helicopter hovering height and/or the position of the spot-gun nozzles relative to the helicopter’s downwash.

The spot-gun method was conceived for use in pest eradication programmes in sensitive areas with specific targeted hosts (small areas or individual host trees). For eradication purposes the spot-gun method is important because it provides maximum pesticide coverage with very little impact to non-host species or the environment. The spot-gun method limits drift by using larger droplet sizes and by using the aircraft’s downwash to provide the foliar coverage needed for efficacy. Large drops from a conventional broadcast boom would not provide enough coverage to achieve efficacy.

This study has provided results that demonstrate that the aerial spot-gun application method has potential to deliver an even distribution of active ingredient onto the leaf surfaces throughout the canopy. The spot-gun method excelled at evenly distributing dye on both the adaxial and abaxial leaf surfaces. A critical next step for validating the spot-gun methodology is to determine if the uneven distribution of dye found at the base of canopy, compared to the top and middle of the canopy, can be mitigated with method adjustment. We did not measure off-target spray drift during this pilot study, however the overall pesticide loading in the target zone is much lower for the spot-gun method compared to that for the broadcast treatment (sum of total active applied in spot treatments/total applied in broadcast treatment), therefore the potential for drift is dramatically reduced. In addition, the drop size used in the spot treatment was coarse with less spray available for drift compared to the smaller drops used in the broadcast method. Further work is needed to quantify the reduction in spray drift produced by the spot-gun method compared to the standard broadcast method and to obtain a dataset for use in aerial spray models.

While there is still significant work to be done to validate and optimise this aerial spot-spray approach, the basic technique (with minor variations) was recently used in what appears to have been a successful eradication of the eucalyptus leaf beetle (*Paropsisterna beata*) that was found in a small number of tall eucalypt trees near Wellington, New Zealand. The organisation in charge of New Zealand’s biosecurity and eradication programmes reported that the method was fast, efficient, and caused minimal disruption to nearby property owners (personal communication).

## Authors’ original submitted files for images

Below are the links to the authors’ original submitted files for images. Authors’ original file for figure 1 Authors’ original file for figure 2 Authors’ original file for figure 3 Authors’ original file for figure 4
