# Supplementary figures and images for: An aerial spot-spraying technique: a pilot study to test a method for pest eradication in urban environments
Source: Springerplus. 2014 Dec 18;3:750. doi: 10.1186/2193-1801-3-750 (PMC4320204; doi:10.1186/2193-1801-3-750)

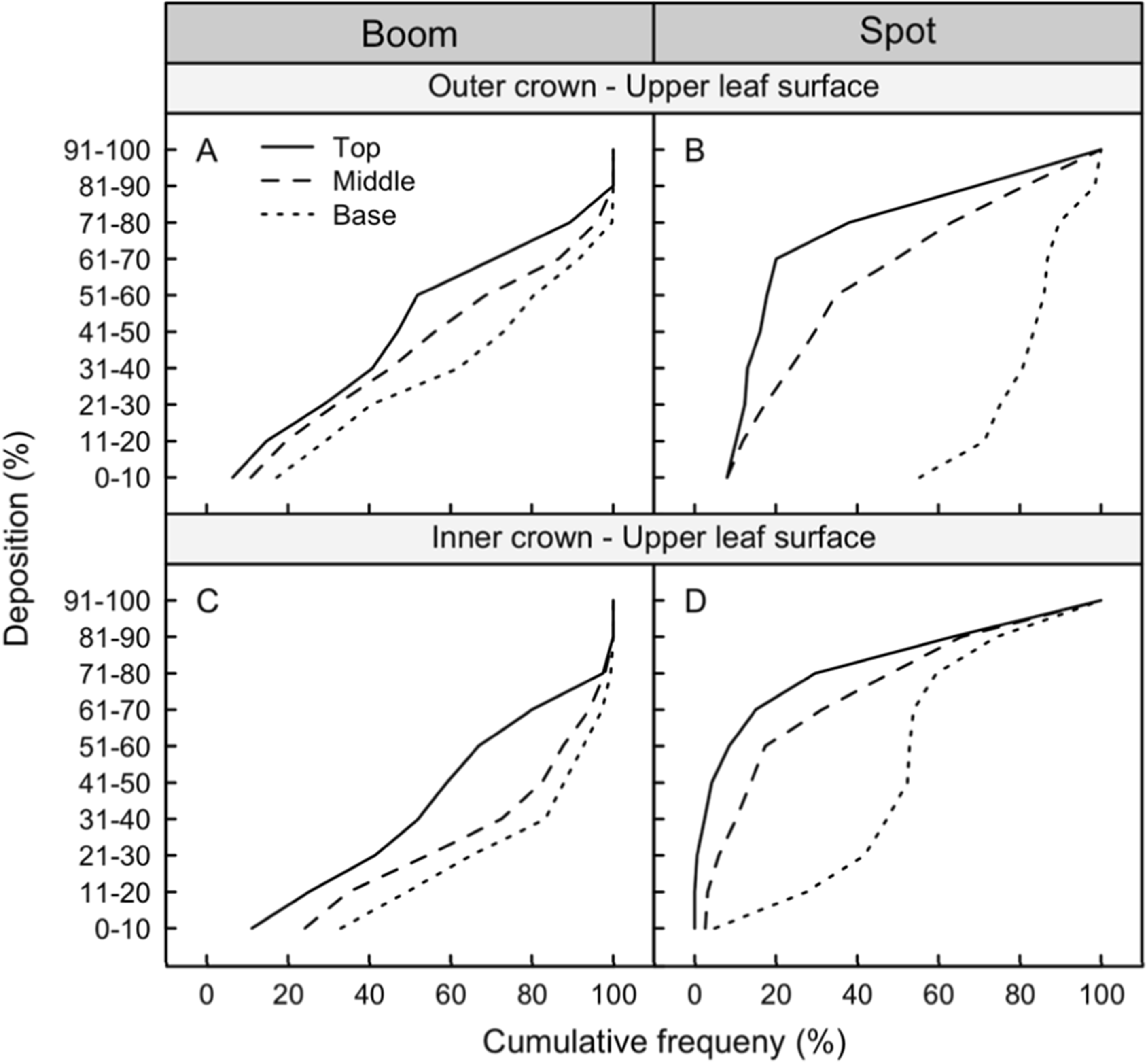

Supplement: Supplementary file 1 — Authors’ original file for figure 1 [file 40064_2014_1524_MOESM1_ESM.tiff]

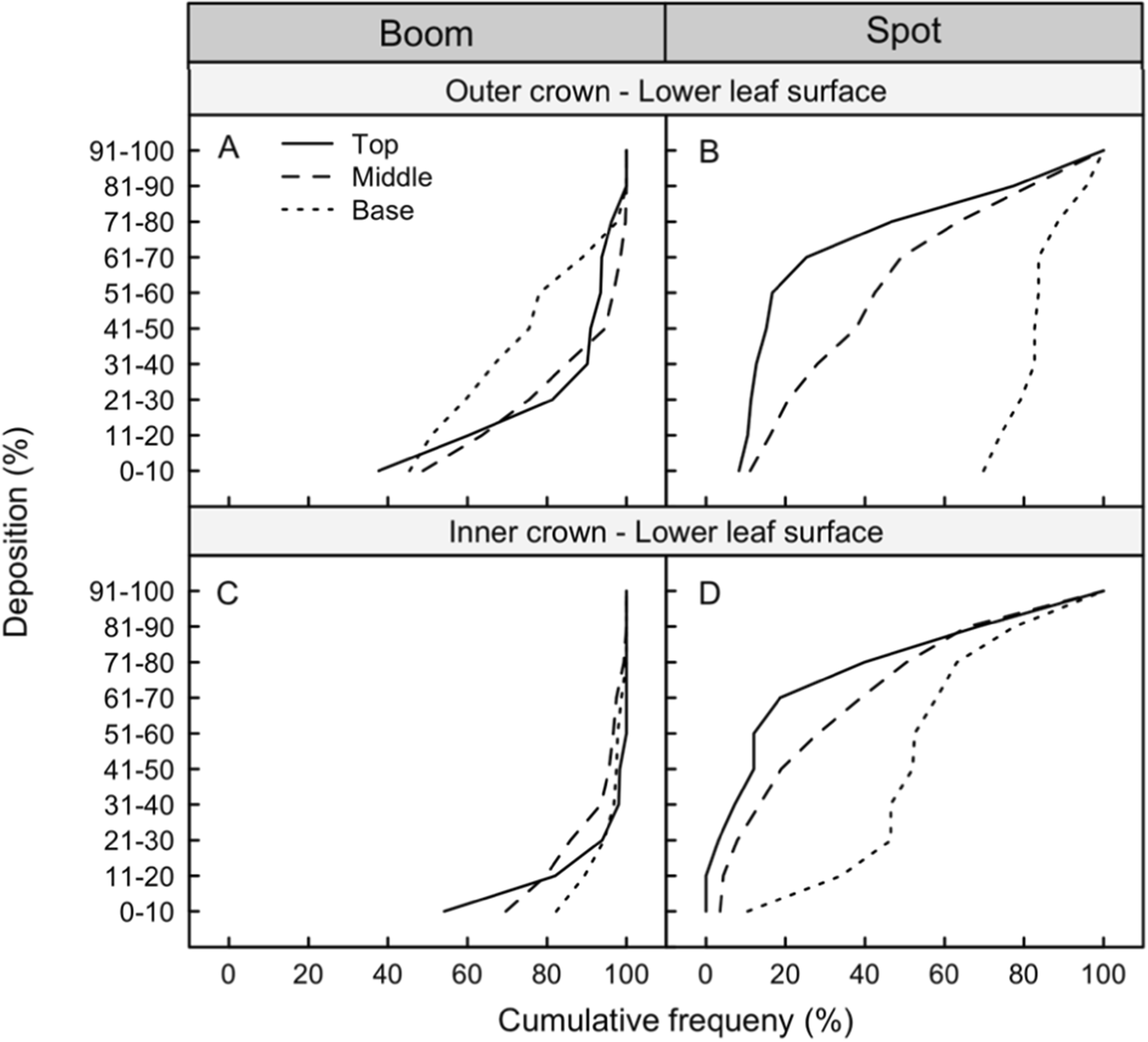

Supplement: Supplementary file 2 — Authors’ original file for figure 2 [file 40064_2014_1524_MOESM2_ESM.tiff]

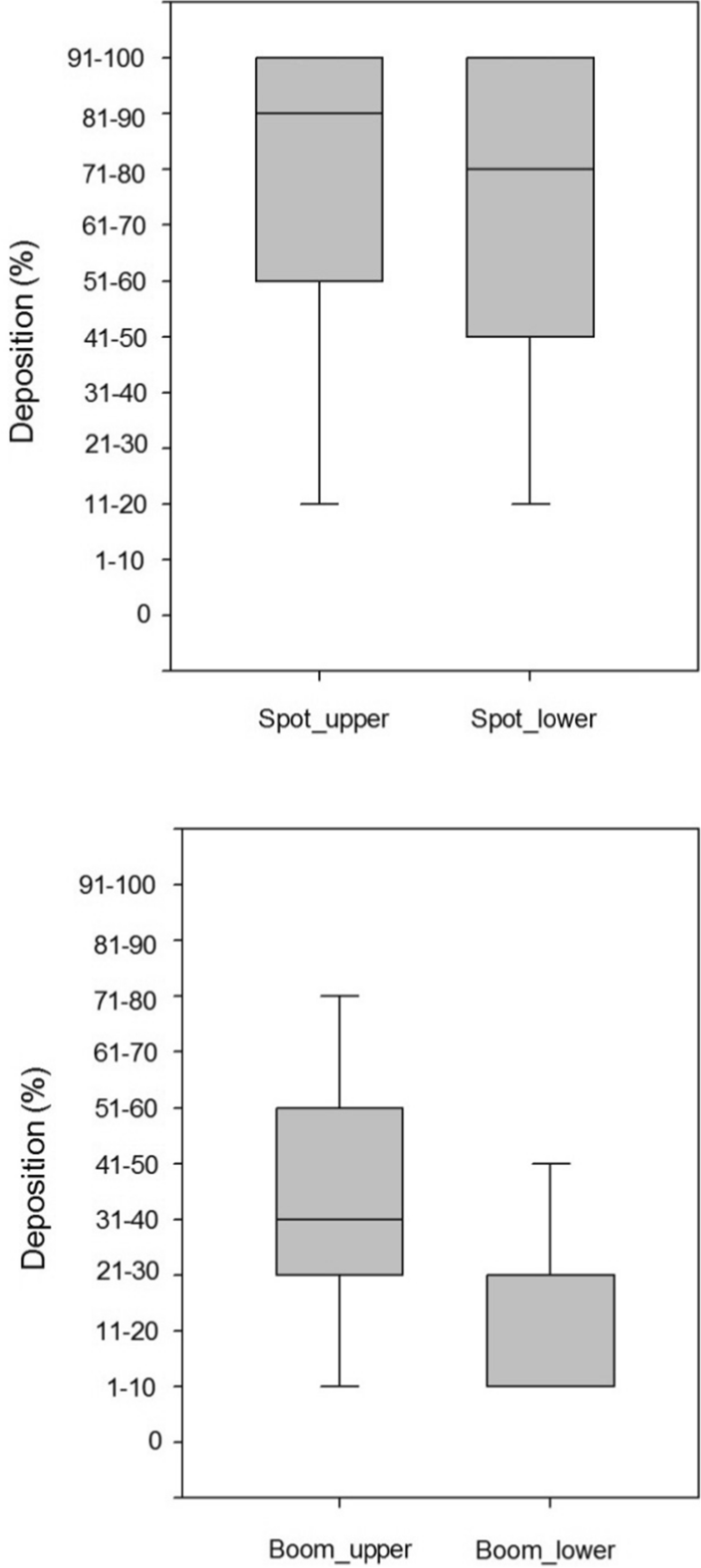

Supplement: Supplementary file 3 — Authors’ original file for figure 3 [file 40064_2014_1524_MOESM3_ESM.tiff]
