# Supplementary material for: An aerial spot-spraying technique: a pilot study to test a method for pest eradication in urban environments
Source: Springerplus. 2014 Dec 18;3:750. doi: 10.1186/2193-1801-3-750 (PMC4320204; doi:10.1186/2193-1801-3-750)
Supplement: Supplementary file 4 — Authors’ original file for figure 4 [file 40064_2014_1524_MOESM4_ESM.pdf]

Spot > 90% - 100%

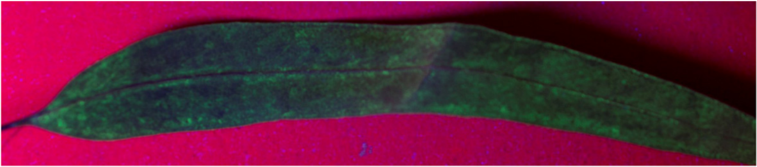

Smear deposit

Boom > 90% - 100%

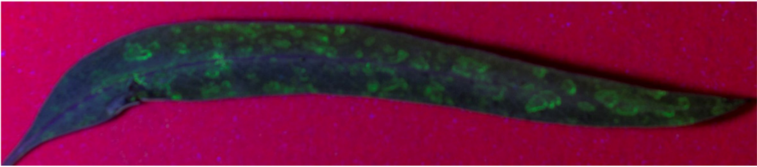

Drop deposit
